# Estimation of internal dose from tap water after the Fukushima Daiichi Nuclear Power Station accident using newly obtained data

**DOI:** 10.1093/jrr/rrz089

**Published:** 2019-12-28

**Authors:** Hirokazu Miyatake, Masaki Kawai, Nobuaki Yoshizawa, Gen Suzuki

**Affiliations:** 1 Mitsubishi Research Institute, Inc., 2-10-3, Nagata-cho, Chiyoda-ku, Tokyo, Japan; 2 International University of Health and Welfare Clinic, 2600–6, Kitakanemaru, Otawara, Tochigi, Japan

**Keywords:** Fukushima Daiichi Nuclear Power Station accident, internal exposure, ^131^I, water intake, thyroid dose

## Abstract

Massive release of radioactive materials into the atmosphere occurred due to the Fukushima Daiichi Nuclear Power Station (FDNPS) accident in March 2011. The World Health Organization (WHO) and the United Nations Scientific Committee on the Effects of Atomic Radiation (UNSCEAR) reported the results of dose estimation to assess the health effect of the accident and both reports state that their assessments of internal and external exposure doses contain certain uncertainties due to uncertainties inherent to the basic data. Therefore, estimation of the internal dose from tap water was conducted in this study by utilizing a database of deposition calculated by an atmospheric transfer, dispersion and deposition model (ATDM) in conjunction with the newly obtained data on the volume of daily water intake obtained by a web-based survey. The median mean and 95-percentile of thyroid equivalent doses were estimated for 1-year and 10-year children and adults in 12 municipalities in the evacuation area in Fukushima prefecture. The present mean thyroid dose estimations for 1-year children (0.4–16.2 mSv) are smaller than the corresponding values in the UNSCEAR 2013 report (1.9–49 mGy). Dose-modifying factors in the Japanese or local community are discussed.

## INTRODUCTION

Large amounts of radionuclides were released into the atmosphere due to the Fukushima Daiichi Nuclear Power Station (FDNPS) accident in March 2011 [1]. According to the United Nations Scientific Committee on the Effects of Atomic Radiation (UNSCEAR) report, the total amount of ^131^I realesed to the atomosphere is estimated at 120 PBq [2]. The World Health Organization (WHO) and UNSCEAR reported the results of dose estimation to assess the health effect of the accident, however both assessments contain certain uncertainties in the basic data or assumptions utilized for dose estimation [2–4]. In this study, the internal dose from tap water was evaluated, as it could be a major source of internal exposure according to a survey of the food supply in the early phase after the accident in Fukushima Prefecture [5].

In our previous study, we used a database of radionuclides deposition calculated by the World-wide version of the System for Prediction of Environmental Emergency Dose Information (WSPEEDI), a kind of atmospheric transfer, dispersion and deposition model (ATDM) simulation program developed by the Japan Atomic Energy Agency (JAEA) National Research and Development Agency that enables users to simulate atmospheric movements, dispersion and disposition of radionuclides and the external exposed dose on a regional to hemisphere scale [6].

In this short communication, we utilized the latest version of WSPEEDI that had improved ATDM simulation by referring to hourly ^137^Cs concentrations in the air measured at many monitoring stations for suspended particulate matter [7, 8]. Additionally, a survey of the volume of water intake for children was undertaken to update the database. Using the new survey data, it was made possible to update the estimation results of internal dose from tap water for the residents in Fukushima Prefecture in the early phase after the accident.

## MATERIALS AND METHODS

### Estimation method for ^131^I concentration in tap water

Since actual ^131^I concentrations in tap water were measured from March 16 or later at only 16 measurement points in Fukushima prefecture, one has to simulate ^131^I concentration in tap water for municipalities where tap water measurements were lacking. The compartment model created by Kawai *et al*. was utilized for the estimation of ^131^I in tap water where it is subject to an increase with every new deposition onto the ground and decays at a fixed rate in relation to the concentration [9].

(1) \begin{equation*} \frac{\mathrm{d}C}{\mathrm{d}t}=a{p}^{\prime }-\lambda C \end{equation*}

*C* is the ^131^I concentration in tap water (Bq/kg), *p*′ is the rate of ^131^I deposition onto the ground, which is calculated by WSPEEDI simulation (Bq/m^2^/h), *a* is the conversion coefficient (m^2^/kg) and $\lambda$ is the effective decay coefficient (1/h).

The conversion coefficient and the effective coefficient are determined by the least square method using the measured values of ^131^I concentration in tap water.

^131^I concentrations in tap water at 16 measurement points in Fukushima prefecture were set according to the previous research [9] which had enough actual measurements to estimate the parameters [10]. In some municipalities there were a few measurement points. In such cases the point names are defined such as Iwaki city 1 and Iwaki city 2 as shown in Table 1. For these 16 points, the concentration was calculated based on the actual measured data. Each conversion coefficient and effective decay coefficient at 16 points and the mean values are shown in Table 1. For other points that had no actual measurements, we estimated the activity concentration of tap water by using the mean value of 16 conversion coefficients and the mean value of 16 effective decay coefficients.

**Table 1:** Conversion coefficients and effective decay coefficients at 16 points

	Municipality	Conversion coefficient	Effective decay coefficient
1	Iwaki city 1	4.0 × 10^−3^	6.0 × 10^−2^
2	Iwaki city 2	4.5 × 10^−4^	3.5 × 10^−3^
3	Ono town	1.7 × 10^−4^	8.4 × 10^−3^
4	Fukushima city	2.1 × 10^−4^	8.6 × 10^−3^
5	Tamura city	2.3 × 10^−4^	5.9 × 10^−3^
6	Minamisoma city 1	1.7 × 10^−4^	6.9 × 10^−3^
7	Minamisoma city 2	5.4 × 10^−4^	1.0 × 10^−2^
8	Iitate village 1	1.7 × 10^−3^	7.5 × 10^−3^
9	Iitate village 2	5.6 × 10^−3^	1.8 × 10^−2^
10	Iitate village 3	5.1 × 10^−3^	2.0 × 10^−2^
11	Date city 1	6.0 × 10^−4^	4.7 × 10^−3^
12	Date city 2	9.2 × 10^−3^	5.9 × 10^−3^
13	Koriyama city 1	5.6 × 10^−4^	8.8 × 10^−3^
14	Koriyama city 2	1.1 × 10^−4^	1.2 × 10^−3^
15	Kawamata town 1	3.6 × 10^−4^	1.3 × 10^−2^
16	Kawamata town 2	3.9 × 10^−4^	1.1 × 10^−2^
	Mean	1.4 × 10^−3^	1.3 × 10^−2^

### Dose estimation from intake of tap water

The effective dose of ^131^I [*D* (Sv)] was estimated using equation (2).

(2) \begin{equation*} D=A\cdotp B\cdotp \Sigma{C}_t \end{equation*}

*A* is the dose coefficient for ^131^I (Sv/Bq), *B* is the volume of daily water consumption (L), *t* is the number of days and *C_t_* is the daily mean concentration of ^131^I in water at *t* (Bq/L).

The dose coefficient is derived from the publications of the International Commission on Radiological Protection (ICRP) [11].

### Volume of daily water intake

In the paper by Ohno *et al*. [12], the volume of water intake is surveyed in detail, showing that the median, arithmetic mean and 95th percentile values are respectively 1.55, 1.65 and 2.91 L/day. There are no major differences in the volume of daily water intake results for 10-year-old children or adults, however, the volume of daily water intake is likely quite different between 1-year-old children and adults where the data are missing. Therefore, a web-based survey on volume of daily water intake for 1-year-old children was conducted targeting mothers nationwide between the ages of 20 years and 49 years with children between the ages of 0 year and 1 year. After approval was obtained from the Institutional Review Boards of the International University of Health and Welfare (approval number: 13-B-277), the survey was conducted from 11 May 2018 to 17 May 2018.

Due to the conditions after the FDNPS accident, it is assumed that juice, bottled tea and water were not available for purchase. Therefore, the amount of potential tap water intake (pTWI) was calculated using the survey data that includes not only tap water but also juice, bottled tea and water used for cooking.

### Evacuation pattern

**Fig. 1:**
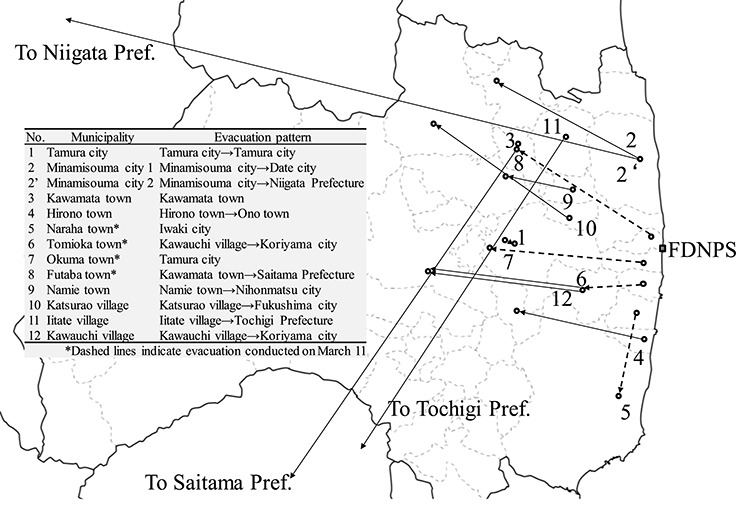
Map of evacuation patterns of municipalities.

The evacuation patterns have been set according to the survey results by Hirakawa *et al*.^5^ as shown in Table 2 and Figure 1 for the residents in each municipality of the evacuation area from March 12 to March 31 [5, 9].

**Table 2:** Representative evacuation pattern of each municipality

No.	Municipality	Evacuation pattern
1	Tamura city	March 12: Hikifune junior high school in Tamura city→March 14: General gymnasium in Tamura city
2	Minamisouma city 1	March 12: Ishigami-daini elementary school in Minamisouma city→March 15: Somegawa gymnasium in Date city
2′	Minamisouma city 2	March 12: Haramachi Ward, Minamisouma city→March 23: Niigata Prefecture
3	Kawamata town	March 12: Iki-iki-sou in Kawamata town
4	Hirono town	March 12: Elder care facility in Hirono town→March 15: Gymnasium in Ono town
5	Naraha town	March 12: Kusano junior high school in Iwaki city
6	Tomioka town	March 12: Kawauchi elementary school →March 16: Big Palette Fukushima in Koriyama city
7	Okuma town	March 12: Denso East Japan in Tamura city
8	Futaba town	March 12: Kawamata elementary school→March 19: Saitama Prefecture
9	Namie town	March 12: Tsushima Activation Center in Namie town→March 15: Towa Culture Center in Nihonmatsu city
10	Katsurao village	March 12: Midori-sou, Community Welfare Center in Katsurao village→March 15: Azuma Gymnasium Park in Fukushima city
11	Iitate village	March 12: Kusano, Iitate village→March 16–21: Tochigi Prefecture and Did not evacuate
12	Kawauchi village	March 12: Kawauchi elementary school→March16: Big Palette Fukushima in Koriyama city

## RESULTS

### Survey results of the volume of daily water intake

**Fig. 2:**
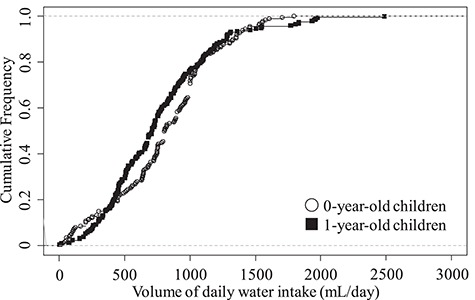
Cumulative frequency of volume of daily water intake (mL/day) of 0- and 1-year-old children.

Survey results were obtained from about 200 mothers with 0- or 1-year-old children. Based on the results, the values of pTWI for each respondent were calculated. Figure 2 shows the cumulative frequency distribution of pTWI.

According to the results of the previous study [12], the 95th percentile for adults was 2.91 L/day. Therefore, when calculating the pTWI of 0- and 1-year-old children, we excluded data that deviated significantly from 2.91 L/day. Comparing the data of 0-year-old children and the data of 1-year-old children by the Mann-Whitney U test, there was no statistically significant difference. Therefore, 0.708, 0.760 and 1.55 L/day were adopted as median, arithmetic mean and 95th percentile of volume of daily water intake of 1-year-old children.

### Dose estimation

Based on the results obtained so far, Table 3 shows the calculated internal exposure doses of ^131^I from tap water. For the dose coefficient for ^131^I (*A* (Sv/Bq)), the values of 4.30$\times$10^−7^, 1.00$\times$10^−6^ and 3.60$\times$10^−6^ Sv/Bq were adapted for adults, 10-year-old children and 1-year-old children, referencing the ICRP report [11]. The mean thyroid equivalent doses of 1-year-old-children ranged from 0.4 to 16.2 mSv, and the highest value was observed in Iitate village.

**Table 3:** Estimated internal exposure dose of ^131^I from tap water (mSv). (a) Median value of water intake, (b) arithmetic mean of water intake, (c) 95 percentile of water intake

Age		Adult			10-year old			1-year old		
Water intake (mL/day)		(a)	(b)	(c)	(a)	(b)	(c)	(a)	(b)	(c)
		1546	1653	2913	1546	1653	2913	708.1	759.8	1538
1	Tamura city	1.0	1.1	1.9	2.4	2.6	4.5	4.0	4.2	8.6
2	Minamisoma city 1	0.4	0.4	0.8	0.9	1.0	1.8	1.6	1.7	3.4
2′	Minamisoma city 2	1.3	1.3	2.4	2.9	3.1	5.5	4.8	5.2	10.5
3	Kawamata town	1.0	1.0	1.8	2.3	2.4	4.3	3.8	4.0	8.2
4	Hirono town	0.7	0.8	1.3	1.6	1.8	3.1	2.7	2.9	5.9
5	Naraha town	0.8	0.9	1.5	1.9	2.0	3.5	3.1	3.3	6.7
6	Tomioka town	1.2	1.3	2.3	2.8	3.0	5.3	4.6	5.0	10.1
7	Okuma town	1.0	1.1	1.9	2.4	2.6	4.5	4.0	4.2	8.6
8	Futaba town	0.7	0.7	1.3	1.6	1.7	3.0	2.6	2.8	5.6
9	Namie town	0.6	0.6	1.1	1.4	1.5	2.7	2.3	2.5	5.0
10	Katsurao village	0.1	0.1	0.2	0.2	0.3	0.5	0.4	0.4	0.9
11	Iitate village	3.9	4.2	7.4	9.2	9.8	17.3	15.1	16.2	32.9
12	Kawauchi village	1.2	1.3	2.3	2.8	3.0	5.3	4.6	5.0	10.1

## DISCUSSION

In the present study, thyroid equivalent doses via tap water ingestion are re-evaluated utilizing new data, i.e. tap water intake volumes based on a nationwide survey and revised ^131^I depositions at water sources estimation by WSPEEDI simulation. In the previous study, only the median doses of municipalities were estimated, whereaas in the present study, median, mean and 95-percentile doses were estimated. If compared, the median thyroid equivalent doses of 1-year-old-children became smaller for most evacuation patterns except for Hirono town and Katsurao village.

In the previous study, the median daily tap water intake was assumed to be 1.8 L/day for adult and 10-year-old non-evacuee residents, and 1 L/d for 1-year-old children [9]. In the present study, the corresponding median values of daily tap water intake were 1.55 and 0.708 L/day, respectively. As 0.6 L/day of bottled water was provided for adult and 10-year-old evacuees, the volume difference between previous and present studies is not large. On the other hand, the water intake volume of 1-year-old children is reduced substantially in the present study.

In order to evaluate the impact of WSPEEDI revision on ingestion dose estimation, the volume of daily water intake is arbitrarily set as in the previous study. Using the same volume of daily water intake as the previous study, there was a difference of about −50% to +10% between the previous result and the present result. This indicates that, even if the WSPEEDI database changes, the estimated result do not change so much. In our model, calculation is performed using the actual measurement value and the estimated WSPEEDI value. Where there is no actual measurement value, the uncertainty is reduced by using the average value of the conversion coefficient, and the effective decay coefficient is obtained using the actual measurement value. Therefore, with the conversion coefficient and effective decay coefficient corrected, even if the estimated value of ^131^I deposition at the water source by WSPEEDI changes, the conversion coefficient and effective decay coefficient are corrected so as to fit actual measurements in 16 points, so the final estimate value of ^131^I concentration of tap water does not change much, indicating the robustness of our one compartment model.

The paper by Kudo. *et al*. shows that a thyroid clearance rate of 30% in the ICRP thyroid model is larger than the actual value of 20% in Japanese volunteers, whereas thyroid volume in the Japanese volunteers is not different from that in the ICRP reference man [13]. Therefore, actual ^131^I activity concentration in the thyroid after a unit dose ingestion will be two-thirds or 0.667 of the ICRP thyroid model in Japanese people. If this is the case, the estimated figures shown in Table 3 could be reduced by two-thirds.

**Fig. 3:**
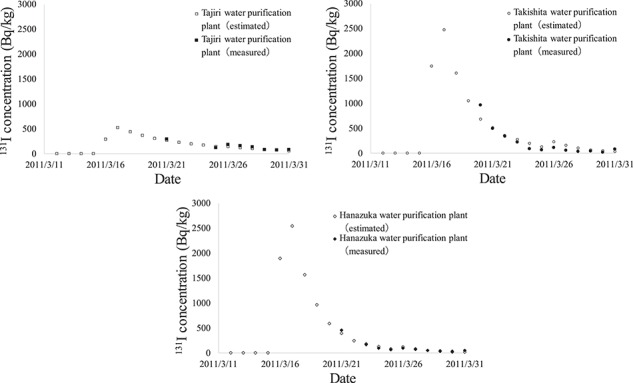
Time series change of the radioactivity concentration of ^131^I in three water purification plants in Iitate village.

The thyroid dose in Iitate village in Table 3 was calculated under the assumption that people evacuated to the Kusano elementary school gymnasium (Table 2), whose water supply was from the Takishita water purification plant. There are three main water purification plants in Iitate village with different contamination levels. Figure 3 shows the time series change of the radioactivity concentration of ^131^I in three water purification plants in Iitate village. The values of the Takishita water purification plant generally show larger values than the others, therefore it can be seen that the value for Iitate shown in Table 3 (based on data from the Takishita water purification plant) is conservative. The supply capacities of the three water purification plants in this village are 395 m^3^/day (Tajiri water purification plant), 1000 m^3^/day (Takishita water purification plant) and 385 m^3^/day (Hanazuka water purification plant) [14, 15]. Comparing the total value of the weighted average, calculated based on these three values, with the total value only for the Takishita water purification plant, the former is about 20% lower than the latter. This result reveals that the rate of Iitate village water supply fluctuation is around 20% considering differences in the water purification plants (i.e. differences in evacuation sites).

In addition to the difference in supply capacity from each water purification plant in Iitate village, it is also necessary to consider coverage of the water supply system in Iitate village. As the coverage of the water supply system in Iitate village is estimated to be 62% in 2010 [15], 38% of people in Iitate village were consuming well water or spring water assumed to be free of radioactive material contamination. In order to correct for this difference, it is necessary to multiply the dose estimation result by a factor of 0.62. Therefore, if we adopt both the weighted average of ^131^I concentrations from the three water purification plants and the coverage rate of the water supply system, the estimated values for Iitate in Table 3 may fall by about 50%, i.e. 0.8 x 0.62 = 0.496. In addition, if an iodine clearance rate of 20% in Japanese people is assumed, the thyroid doses of Iitate residents could be reduced by a further two-thirds and would be one-third of the estimates in Table 3.

Finally, it is difficult to compare estimated thyroid doses between those in the UNSCEAR 2013 report (Table C-18.4, 18.5, and 18.6) and those in the present study (Table 3), since the former doses are from tap water and other food stuff, while the latter doses are from tap water only. If tap water was the major route of dose ingestion after the FDNPS accident, as Hirakawa *et al*. pointed out [5], the present thyroid dose estimations for 1-year children (0.4–16.2 mSv) are smaller than the corresponding values in the UNSCEAR 2013 report (1.9–49 mGy).

## CONCLUSION

In conclusion, by revising the volume of daily water intake as well as the estimated ^131^I depositions on water sources by WSPEEDI, the median, mean and 95-percentile values of thyroid equivalent doses were estimated. Estimated thyroid doses in the present study are smaller than those in our previous study in most municipalities except for Hirono town and Katsurao village. In addition, thyroid dose modifying factors in Japanese or local communities are discussed for realistic dose estimation.

## References

[1] WHO Preliminary dose estimation from the nuclear accident after the 2011 great East Japan earthquake and tsunami. 2012.

[2] UNSCEAR Annual report: Sources, effects and risks of ionizing radiation UNSCEAR 2013, report, volume I, report to the general assembly scientific annex a: Levels and effects of radiation exposure due to the nuclear accident after the 2011 great East-Japan earthquake and tsunami. 2013.

[3] WHO Health risk assessment from the nuclear accident after the 2011 great East Japan earthquake and tsunami, based on a preliminary dose estimation. 2013.

[4] Terada H, Chino M Improvement of worldwide version of system for prediction of environmental emergency dose information (WSPEEDI), (II), J. Nucl. Sci. Technol. 2014: 41(5), 632–640

[5] Hirakawa S, Yoshizawa N, Murakami K et al. Surveys of food intake just after the nuclear accident at the Fukushima Daiichi nuclear Power Station, J. Food Hyg. Soc. Jpn. 2017; 58(1):36–42. 10.3358/shokueishi.58.36 28260731

[6] Nagai H, Terada H, Tsuduki K et al. Updating source term and atmospheric dispersion simulations for the dose reconstruction in Fukushima Daiichi nuclear Power Station accident. EPJ Web of Conferences 2017;153:08012.

[7] Oura Y, Ebihara M, Tsuruta H et al. Database of hourly atmospheric concentrations of Radiocesium (^134^Cs and ^137^Cs) in suspended particulate matter collected in march 2011 at 99 air pollution monitoring stations in eastern Japan. J. Nucl. Radiochem. Sci. 2015;15:15–26.

[8] Terada H, Nagai H, Tsuduki K et al. Refinement of source term and atmospheric dispersion simulations of radionuclides during the Fukushima Daiichi nuclear Power Station accident. J. Environ. Radioactiv 2019; (submitted). 10.1016/j.jenvrad.2019.106104 31983441

[9] Kawai M, Yoshizawa N, Suzuki G ^131^I dose estimation from intake of tap water in the early phase after Fukushima Daiichi nuclear power plant accident, Radiation protection dosimetry 2017; 179(1): 43–48. 10.1093/rpd/ncx208 29618130

[10] Ministry of Health, Labour and welfare (in Japanese): http://www.mhlw.go.jp/file/06-Seisakujouhou-10900000-Kenkoukyoku/1_houshasei_110719_m1.pdf. (7 August 2019, date last accessed).

[11] ICRP Compendium of Dose Coefficients based on ICRP Publication 60, Vol. 119 ICRP Publication, 2012. 10.1016/j.icrp.2012.06.038 23025851

[12] Ohno K, Asami M, Matsui Y. Is the default of 2 liters for daily per-capita water consumption appropriate? A nationwide survey reveals water intake in Japan. J. Water Health 2018; 16 (4): 562–573. 30067239 10.2166/wh.2018.281

[13] Kudo T, Inano A, Midorikawa S et al. Determination of the kinetic parameters for ^123^I uptake by thyroid, and thyroid weights and volumes, in present-day healthy Japanese volunteers. Health Physics Journal 2019; in press. 10.1097/HP.0000000000001144 32015244

[14] Iitate village: The water supply business water-quality test plan of 2016 (Report in Japanese). http://www.vill.iitate.fukushima.jp/uploaded/attachment/4095.pdf (7 August 2019, date last accessed).

[15] Fukushima prefecture: Water supply of Fukushima (2011) (Report in Japanese). https://www.pref.fukushima.lg.jp/uploaded/attachment/52438.pdf (7 August 2019, date last accessed).

